# Prostacyclin reverses the cigarette smoke-induced decrease in pulmonary Frizzled 9 expression through miR-31

**DOI:** 10.1038/srep28519

**Published:** 2016-06-24

**Authors:** M. A. Tennis, M. L. New, D. G. McArthur, D. T. Merrick, L. D. Dwyer-Nield, R. L. Keith

**Affiliations:** 1 University of Colorado Denver, Aurora, Colorado, USA; 2 Denver Veterans Administration Medical Center, Denver, Colorado, USA.

## Abstract

Half of lung cancers are diagnosed in former smokers, leading to a significant treatment burden in this population. Chemoprevention in former smokers using the prostacyclin analogue iloprost reduces endobronchial dysplasia, a premalignant lung lesion. Iloprost requires the presence of the WNT receptor Frizzled 9 (Fzd9) for inhibition of transformed growth *in vitro*. To investigate the relationship between iloprost, cigarette smoke, and Fzd9 expression, we used human samples, mouse models, and *in vitro* studies. Fzd9 expression was low in human lung tumors and in progressive dysplasias. In mouse models and *in vitro* studies, tobacco smoke carcinogens reduced expression of Fzd9 while prostacyclin maintained or increased expression. Expression of miR-31 repressed Fzd9 expression, which was abrogated by prostacyclin. We propose a model where cigarette smoke exposure increases miR-31 expression, which leads to decreased Fzd9 expression and prevents response to iloprost. When smoke is removed miR-31 is reduced, prostacyclin can increase Fzd9 expression, and progression of dysplasia is inhibited. Fzd9 and miR-31 are candidate biomarkers for precision application of iloprost and monitoring of treatment progress. As we continue to investigate the mechanisms of prostacyclin chemoprevention and identify biomarkers for its use, we will facilitate clinical trials and speed implementation of this valuable prevention approach.

Lung cancer continues to be the leading cause of cancer death in the United States, with most cancers diagnosed in former smokers. While avoidance of tobacco abuse is clearly the strongest deterrent of lung cancer, chemoprevention in former smokers may be a more effective intervention than treatment of established lung cancers. Former smokers treated with iloprost in a phase II clinical trial had improved endobronchial dysplasia (a precancerous lung lesion)^1^. This trial demonstrated that dysplastic lesions in the lung can be targets of chemoprevention and, due to the presence of fewer abnormalities, may be more responsive than tumors to treatments aimed at halting progression. Iloprost is a prostacyclin analogue and prostacyclin is associated with maintaining a normal lung epithelium^2^. Increased prostacyclin in mouse models, through genetic overexpression of prostacyclin synthase (PGIS) or treatment with iloprost, reduces the development of lung cancer after urethane or cigarette smoke exposure^3^,^4^. Most non-small cell lung cancer (NSCLC) expresses no or very low levels of PGIS and the prostaglandin I2 (prostacyclin) receptor (IP). When IP is knocked out in a mouse model combined with PGIS overexpression, these mice are still protected from lung cancer, suggesting that prostacyclin does not require IP for chemoprevention in the lung^4^. Prostacyclin activates peroxisome proliferator activated receptor gamma (PPARγ) and lung specific overexpression of PPARγ reduces urethane induced tumor multiplicity^5^,^6^. Overexpression of PPARγ combined with iloprost did not improve lung tumor prevention over iloprost alone, indicating that PPARγ is a critical target of iloprost activated signaling^4^.

As a known activator of PPARγ, Frizzled 9 (Fzd9) was proposed as an alternative receptor for prostacyclin in the lung. Fzd9 is a G-protein coupled transmembrane receptor that is required for the tumor suppressive activity of the Wnt7a signaling pathway in the lung. Binding to Fzd9 by its endogenous ligand Wnt7a leads to increased PPARγ activity and maintenance of a normal lung epithelium^7^,^8^. We demonstrated that Fzd9 is required for iloprost activation of PPARγ and transformed growth inhibition *in vitro*^9^. Loss of Fzd9 could be an early event in the development of lung cancer, perhaps even in pre-malignant lesions, and might prevent response to iloprost. Of the 49 patients in the iloprost chemoprevention trial who received iloprost, 23 had regressive histology and 25 had stable or progressive histology^1^. This presents an opportunity for distinguishing responders in a high-risk population, which may be facilitated by measuring Fzd9 expression in biopsies. The effect of iloprost and cigarette smoke on Fzd9 expression in NSCLC cell lines is negligible, but their effect on Fzd9 in normal bronchial epithelial cells has not been investigated^9^. We also have little understanding of how Fzd9 expression is regulated or lost in the lung. In this study, we measured Fzd9 expression in human normal and tumor lung tissues, dysplastic cell lines, and Human Bronchial Epithelial Cells (HBEC) and examined the effects of cigarette smoke and iloprost on Fzd9 expression. We also investigated the role of miR-31 in determining how cigarette smoke and iloprost regulate Fzd9 expression.

## Results

### Fzd9 expression is decreased in lung tumors and lung dysplasia

**Figure 1:**
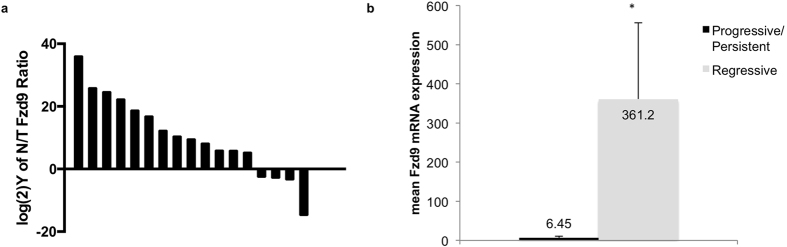
Lung tumors and persistent/progressive dysplasias have decreased expression of Fzd9. (**a**) Matched pairs of human normal and tumor lung tissue were analyzed for Fzd9 expression by qPCR. Data is presented as the log(2)Y of normal/tumor Fzd9 and each bar is one tissue pair. Positive values represent pairs with higher Fzd9 in the normal sample and negative values represent pairs with higher Fzd9 in the tumor. (**b**) Cultured cells from dysplastic biopsies were analyzed for Fzd9 expression by qPCR. Cell lines are grouped by clinical designation as regressive or progressive/persistent. Bars represent standard error of the mean. *p = 0.05.

To determine whether Fzd9 expression changes in human lung cancer, we measured Fzd9 mRNA levels in matched normal and tumor lung tissue. Seventy-five percent (13/17 pairs) of patient samples had higher Fzd9 expression in uninvolved lung tissue compared to tumor tissue. Higher Fzd9 in normal tissues resulted in normal to tumor Fzd9 expression ratios greater than 1 (positive with log(2)Y transformation), while higher Fzd9 in the tumor tissue led to normal to tumor ratios of less than 1 (negative with log(2)Y transformation) (Fig. 1a). We previously characterized dysplastic cells collected from repeat endobronchial biopsies and identified dysplasias that regressed, persisted, or progressed based on histologic grade^10^. In cultures from these cells, Fzd9 expression is higher in regressive dysplasias compared to persistent and progressive dysplasias (Fig. 1b). These data suggest that Fzd9 expression is frequently decreased in human NSCLC, is down-regulated early in progression, and may have a role in maintaining normal lung epithelium and preventing cancer development.

### Tobacco smoke carcinogens decrease and prostacyclin increases Fzd9 expression *in vivo*

**Figure 2:**
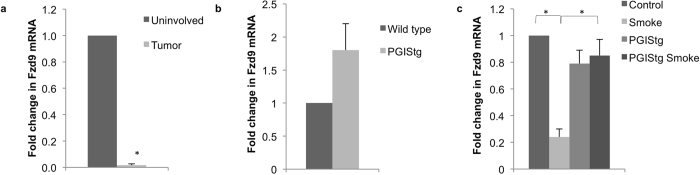
Fzd9 expression *in vivo* is decreased with tobacco carcinogen exposure and maintained by prostacyclin. Fzd9 mRNA levels were determined by qPCR. (**a**) AJ mice received a single intraperitoneal injection of saline or urethane at 1 mg/g and were sacrificed 20 weeks later. Uninvolved lung tissue and tumors were dissected for RNA extraction. *p = 0.003 (**b**) FVB wild type and transgenic mice were exposed to urethane and sacrificed 20 weeks later. Tumors were dissected out of the lungs for RNA extraction. p = 0.06 (**c**) FVB wild type and PGIS transgenic mice were exposed to one week of cigarette smoke. Whole lung was removed for RNA extraction. Control vs Smoke, p = 0.0005; Smoke vs PGIStg Smoke, p = 0.0002. Bars represent standard error of the mean. Data is normalized to Rpl13a. PGIStg, prostacyclin synthase transgenic.

We measured Fzd9 mRNA expression by qPCR in tumors from AJ mice treated with one dose of urethane and sacrificed after 20 weeks. Urethane typically induces 30 lung tumors/mouse in AJ mice and 8–10 in FVB mice. In AJ mice, Fzd9 expression decreased in urethane induced tumor tissue compared to uninvolved matched lung tissue (Fig. 2a). PGIS overexpression protects mice from urethane induced lung tumors, reducing tumor burden by 50%^11^. In a urethane model using FVB PGIStg mice, PGIS overexpression leads to increased prostacyclin levels and decreased tumor burden^11^. Fzd9 expression in urethane-induced tumors from these mice is higher in PGIStg compared to wild type (Fig. 2b). This correlates with previously published data demonstrating increased Fzd9 expression in mice receiving iloprost after urethane^9^. To examine the earliest changes in smoke exposed PGIStg mice, we exposed these mice to one week of cigarette smoke at 80 mg/m^3^. In whole lungs from this model, smoke exposed mice had decreased expression of Fzd9 compared to unexposed controls (Fig. 2c). In smoke-exposed PGIS overexpressing mice, Fzd9 expression remains higher compared to wild type smoked mice, suggesting that prostacyclin helps to maintain expression of Fzd9 after a carcinogenic insult (Fig. 2c). The analyses in whole lung from our mouse models may be confounded by the complexity of the cell mixture in whole lung. While isolation of epithelial cells would more definitively assign expression to the epithelium, testing of Fzd9 gene expression in human clinical trials would likely be done by lung biopsy, which also contains a mixed cell population.

### Prostacyclin and cigarette smoke affect Fzd9 expression in cultured human bronchial epithelial cells

**Figure 3:**
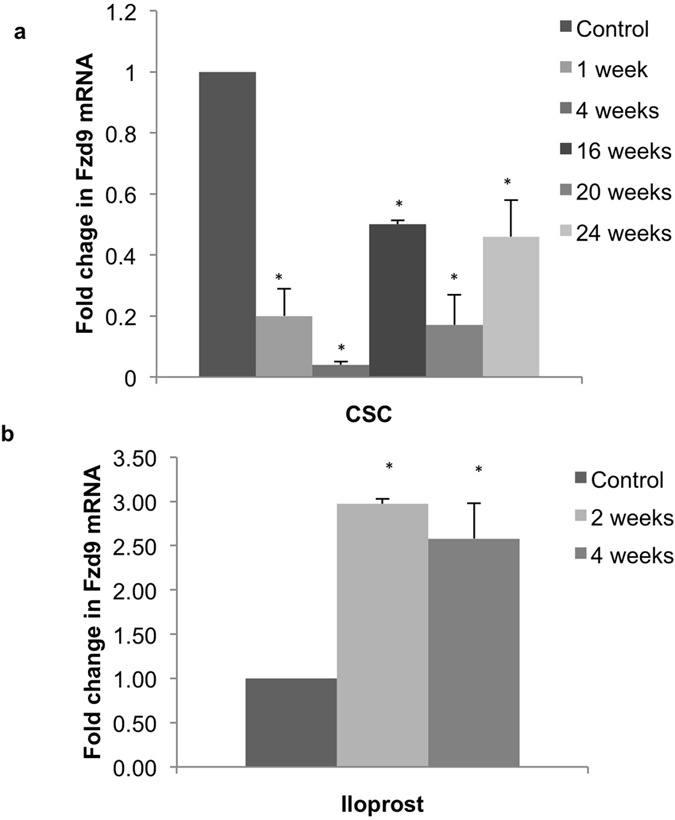
Fzd9 expression is altered by CSC and iloprost *in vitro*. HBEC were treated in triplicate with iloprost or CSC, mRNA was extracted, and Fzd9 mRNA was measured in triplicate by qPCR. (**a**) 1 to 24 weeks of 5 ug/ml twice weekly CSC compared to unexposed controls. (**b**) 10 um iloprost twice weekly for 2 and 4 weeks compared to unexposed controls. Bars represent standard error of the mean. CSC, cigarette smoke condensate. *mean vs control p < 0.05.

**Figure 4:**
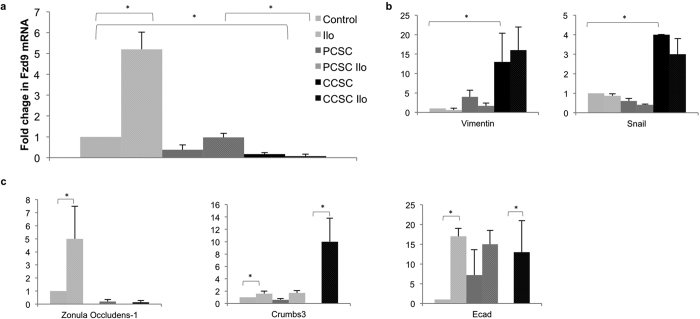
Fzd9 expression is increased by iloprost in previously CSC exposed cells. (**a**) In a 20-week model, HBEC were treated with 16 weeks of CSC followed by 4 weeks of 10 uM iloprost, vehicle, and/or CSC. Control vs Ilo p = 0.03 Control vs PCSC p = 0.08, Control vs CSC p = 0.007, PCSC vs PCSC ilo p = 0.1, PCSC vs CCSC p = 0.1, PCSC ilo vs CCSC ilo p = 0.002. Legends in b and c are identical to a. (**b**) mRNA expression of mesenchymal genes in the 20-week model. Vim Control vs CSC p = 0.04, Snail Control vs CSC p = 0.02. (**c**) mRNA expression of epithelial genes in the 20 week model. ZO1 control vs ilo p = 0.007, CRB3 PCSC vs PCSC Ilo p = 0.04, CRB3 CSC vs CSC ilo p = 0.008, Ecad Control vs Ilo p = 0.007. All data is normalized to GAPDH. Bars represent standard error of the mean. Ilo, iloprost; PCSC, previous cigarette smoke condensate; CCSC, current cigarette smoke condensate.

We were interested in developing an *in vitro* model of chemoprevention using HBEC, cigarette smoke condensate (CSC), and iloprost in order to elucidate mechanisms involved in prostacyclin chemoprevention. W measured Fzd9 mRNA by qPCR at multiple CSC and iloprost exposure time points to investigate the relationship between Fzd9, cigarette smoke, and prostacyclin. The first culture of HBEC was split into three plates for CSC or iloprost and three plates for control. Plates were then carried and exposed continuously for the indicated time points. In HBEC, Fzd9 expression decreased below baseline at all times points after CSC exposure (Fig. 3a). Treatment of HBEC with iloprost for 2 and 4 weeks increased Fzd9 expression (Fig. 3b). To mimic the human condition of former and current smoking, we treated HBEC with CSC or dimethyl sulfoxide (DMSO) vehicle for 16 weeks. We then removed CSC exposure (previous CSC or PCSC) or kept CSC exposure (continued CSC or CCSC) and treated with iloprost or DMSO for 4 more weeks, for a total of 20 weeks of continuous culture. In HBEC cells treated with 16 weeks of CSC followed by 4 weeks of iloprost Fzd9 expression increased compared to the control (PCSC ilo vs PCSC) (Fig. 4a). When CSC was removed after week 16 (PCSC), Fzd9 expression was higher than in cells exposed to 20 continuous weeks of CSC (CCSC), though expression did not returned to CSC naive levels (Control) (Fig. 4a). This suggests that withdrawal of cigarette smoke increases expression of Fzd9, but there is a delay in return to normal levels. Continued exposure to cigarette smoke maintains suppression of Fzd9 expression.

Treating HBEC with CSC and iloprost in culture does not lead to measureable morphologic changes like those observed in former smokers’ dyplasias in the oral iloprost clinical trial. Changes in epithelial to mesenchymal transition (EMT) gene expression occur early in the conversion of normal epithelium in smoke exposed lung, so we used EMT gene expression as a surrogate for dysplasia measurement in our HBEC 20 week model^12^. We observed a trend where mesenchymal genes Vimentin and Snail decreased with iloprost treatment in previous CSC exposure (PCSC) and remained higher with current CSC (CCSC) exposure (Fig. 4b). Epithelial genes Zonula Occludens-1, Crumbs3, and Ecadherin tended to increase with iloprost treatment (ilo), decreased with any CSC exposure (PCSC and CCSC), and increased when iloprost was added to previous or current CSC exposed cells (PCSC ilo and CCSC ilo) (Fig. 4c). Iloprost appears to help reverse the mesenchymal phenotype induced by cigarette smoke exposure. These *in vitro* studies using HBEC support a transcriptional relationship between iloprost and Fzd9 that is altered by cigarette smoke but can be restored by removing carcinogen exposure.

### miRNA changes in HBEC with iloprost and CSC

**Figure 5:**
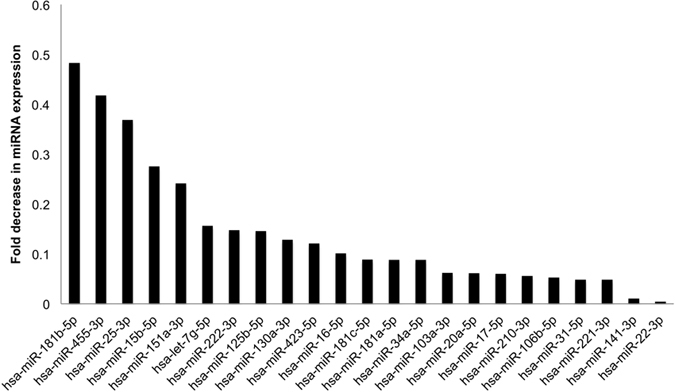
miRNA are altered after iloprost treatment in previously CSC exposed cells. 23 miRNA had decreased expression in an initial screen using a cancer stem cell focused PCR array. Fold decrease is for cells exposed to CSC for 24 weeks followed by 4 weeks of iloprost compared to 4 weeks of DMSO (baseline of 1). miRNA included on this graph passed software quality control and had a greater than two-fold decrease in expression.

Regulation of Fzd9 in the normal lung and during lung cancer progression is poorly understood, so we are interested in exploring mechanisms leading to prostacyclin’s effect on Fzd9. We are also interested in identifying biomarkers for predicting response to iloprost and monitoring drug efficacy during iloprost treatment. As biomarkers, miRNA have strong potential because they are highly stable and exhibit tissue specificity^13^. miRNA expression has been associated with smoke exposure, stages of lung cancer progression, tumor aggressiveness, and treatment resistance^14^,^15^,^16^. A mouse model of lung cancer has demonstrated that chemopreventive agents bexarontene and pioglitazone can modulate miRNA in the context of smoke exposure^17^. miRNA are also linked with EMT regulation, which may be involved in the conversion of stem cells to cancer stem cells^12^. To identify relevant miRNA, we employed a qPCR array to measure miRNA changes *in vitro* after exposure to iloprost. The array was focused on cancer stem cell miRNA, which may affect the earliest changes in lung epithelial cells caused by carcinogen exposure and reversed by iloprost treatment. HBEC cells were exposed to 24 weeks of CSC followed by 4 weeks DMSO or 24 weeks of CSC followed by 4 weeks of iloprost. In these cells, expression of 23 miRNA was decreased with previous smoke exposure and iloprost compared to previous smoke exposure alone (Fig. 5). Several of these miRNA are increased in lung cancer or with smoke exposure (miR-221, miR-106b, miR-25, miR-31, miR-17, and miR-141) or decreased by prostacyclin (miR-221)^18^,^19^,^20^,^21^,^22^.

### miR-31 mediates prostacyclin and cigarette smoke induced Fzd9 expression changes

**Figure 6:**
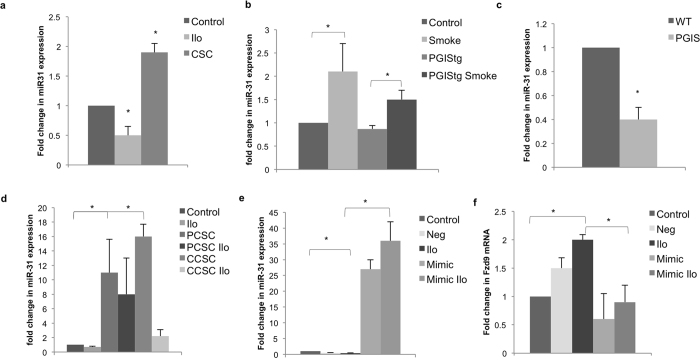
Iloprost increases Fzd9 by reducing miR-31 expression. (**a**) miR-31 expression in HBEC treated with 10 uM iloprost or 5 ug/ml CSC for 4 weeks. *p < 0.04. (**b**) miR-31 expression in FVB wild type and PGIStg mice exposed to cigarette smoke for 1 week. miRNA was extracted from whole lung tissue. Control vs Smoke p = 0.05, PGIStg vs PGIStg Smoke p = 0.01, Smoke vs PGIS Smoke p = 0.1. (**c**) miR-31 expression in whole lung from PGIStg mice compared to wild type mice. p = 0.02. (**d**) In a 20 week model, miR-31 expression was measured in HBEC cells treated with 16 weeks of CSC or vehicle followed by 4 weeks of iloprost, vehicle, and/or CSC. Control vs PCSC p = 0.01, Control vs CSC p = 0.006. (**e**) miR-31 expression in HBEC with six weeks of iloprost treatment after transfection with a miR-31 mimic. Neg vs Mimic p = 0.002, Ilo vs Mimic Ilo p = 0.0004. (**f**) Fzd9 expression in HBEC with six weeks of iloprost treatment after transfection with a miR-31 mimic. Control vs ilo p = 0.02, Neg vs Mimic p = 0.08, Ilo vs Mimic Ilo p = 0.02. miR-31 and Fzd9 levels were measured by qPCR. miR-31 is normalized to RNU6 and Fzd9 to GAPDH. Transfections were done in duplicate. Bars represent standard error of the mean. Ilo, iloprost; CSC, cigarette smoke condensate; PCSC, previous CSC; CCSC, current CSC; Neg, mimic negative control.

We further investigated some of the miRNA that were most strongly affected by the four weeks of iloprost with previous smoke exposure, including miR-221, miR-222, and miR-31. Of these three, individual qPCR for expression changes after CSC and iloprost exposure in HBEC confirmed that miR-31 expression increased with 4 weeks of CSC and decreased with 4 weeks of iloprost (Fig. 6a). Previous studies found that miR-31 expression is increased in NSCLC and associated with shorter overall survival^23^,^24^,^25^. A miR-31 transgenic mouse has increased lung hyperplasia, adenoma, and adenocarcinoma and promotes KRAS mediated oncogenesis by directly reducing expression of negative regulators of RAS/MAPK signaling^26^. miR-31 and let-7 coordinate to maintain balance in proliferation of lung cancer stem-like cells, suggesting a role for miR-31 in the earliest cellular changes leading to lung cancer^27^. We found that miR-31 expression increased with smoke exposure in the one week mouse model and was abrogated with increased prostacyclin levels in PGIStg mice (Fig. 6b). In whole lung from the PGIStg mice compared to wild type mice, miR-31 expression decreased (Fig. 6c). Fzd9 expression was inverse to miR-31 expression in these mouse models (Fig. 2). To examine a potential role for miR-31 in iloprost chemoprevention in former smokers, we measured expression in our 20 week *in vitro* model that exposed HBEC cells to CSC and iloprost (Fig. 6d). Cells were treated with 16 weeks of CSC followed by 4 weeks of DMSO (PCSC) or iloprost (PCSC ilo), CSC (CCSC), or CSC and iloprost (CCSC ilo). miR-31 expression increased in both previous (PCSC) and current CSC (CCSC) exposed cells compared to control cells. miR-31 was slightly lower in previous CSC (PCSC) exposed cells, suggesting the full effect of CSC removal on miR-31 expression required more time without CSC. Iloprost tended to reduce miR-31 expression in both previous (PCSC ilo) and current CSC (CSC ilo) exposure, though it is unclear why miR-31 expression is lower in CCSC ilo compared to PCSC ilo. Fzd9 expression was not increased in currently CSC exposed cells with iloprost (CCSC ilo) (Fig. 4a), suggesting that while mir-31 expression changes with iloprost, it is not the only contributor to Fzd9 repression in the presence of cigarette smoke condensate.

Previous studies suggest that iloprost binds with Fzd9 to induce anti-cancer signaling, however, prostacyclin may also generate effects by increasing Fzd9 expression^9^. Since our data demonstrated an inverse relationship between Fzd9 and miR-31 expression with exposure to iloprost (comparing Figs 2 and 4 with Fig. 6), we hypothesized that miR-31 may be a link between prostacyclin and Fzd9 expression. To determine whether changes in miR-31 affected Fzd9 expression, a miR-31 mimic was transfected into cells with 6 weeks of iloprost treatment. Transfection of a miR-31 mimic resulted in at least 25-fold increased levels of miR-31 in the difficult to transfect HBEC line (Fig. 6e). miR-31 expression induced by mimics was not reduced by the addition of iloprost. Fzd9 expression decreased in the cells with increased miR-31 expression (Fig. 6f). miR-31 mimic expression that could not be overcome by iloprost also resulted in lower Fzd9 levels (Fig. 6f). The presence of high levels of miR-31 prevented the increased Fzd9 expression induced by iloprost, suggesting that miR-31 is a link between iloprost and Fzd9.

## Discussion

In a successful chemoprevention trial, the prostacyclin analogue iloprost decreased endobronchial dysplasia in former smokers. Bronchial dysplasia has long been presumed to be a precursor to squamous cell carcinoma and a recent study used successive bronchoscopies to verify that persistent bronchial dysplasia is, in fact, associated with development of invasive squamous cell carcinoma^10^. Based on this trial and ongoing preclinical studies, prostacyclin has the potential to be a precision medicine approach to lung cancer prevention. To advance the use of prostacyclin chemoprevention, we need to better understand the mechanisms behind the reduction of endobronchial dysplasia by iloprost and identify markers for targeting its application. Similar to personalized lung cancer therapy, only certain lesions will respond to prostacyclin based treatment and we have identified a potential marker for response. Early loss is an important characteristic for markers in cancer prevention. If Fzd9 is important in initial stages of cellular dysfunction, then understanding how Fzd9 expression is lost in lung cancer is key to prevention with an agent that signals through Fzd9. It is critical to complete *in vitro* studies to establish mechanisms and markers prior to investigations in our human samples.

**Figure 7:**
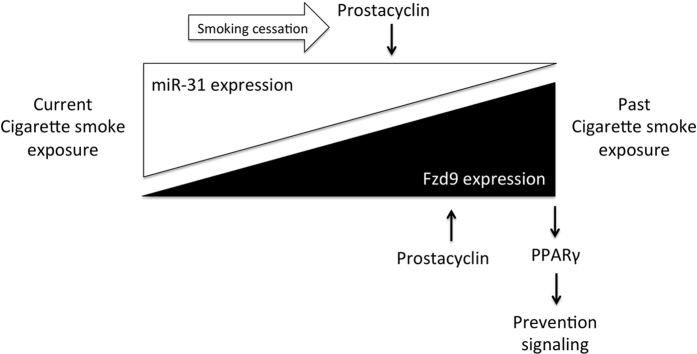
Model of relationships between miR-31, cigarette smoke, and Fzd9. In this model, cigarette smoke increases miR-31 expression, which prevents prostacyclin from increasing Fzd9 expression. When cigarette smoke is removed, prostacyclin is able to reduce miR-31 expression, increase Fzd9 expression, and initiate prevention signaling through PPARγ.

We demonstrated decreased expression of Fzd9 in human lung tumor and dysplastic tissue, suggesting that loss of Fzd9 may contribute to establishment of tumors and that it influences early development of premalignant lesions. Examination of tissues from mouse models of cigarette smoke exposure and *in vitro* studies verified that Fzd9 expression is decreased by both urethane (a tobacco smoke component) and cigarette smoke. Increased prostacyclin in these models leads to increase or maintenance of Fzd9 expression, supporting a relationship between prostacyclin and Fzd9 expression in the context of lung cancer development. If the mouse models parallel the human experience, Fzd9 expression will be lower in cigarette smoke-exposed populations at high risk for lung cancer. We propose a model where cigarette smoke exposure decreases Fzd9 expression, in part by increasing miR-31 and preventing normal signaling between prostacyclin and Fzd9. When cigarette smoke exposure is removed, prostacyclin reduces miR-31 expression and Fzd9 expression begins to return (Fig. 7).

While tumors had increased Fzd9 expression in mice with increased prostacyclin levels, iloprost treatment does not increase Fzd9 expression in NSCLC cell lines^9^. This could be due to differences between PGIS overexpression, with continuously high prostacyclin levels present from birth, and treatment with iloprost, a lower dose of prostacyclin. Alternatively, NSCLC cell lines contain more genetic and epigenetic alterations than single-agent induced mouse tumors and thus may be unable to respond to iloprost due to increased aberrations. In CSC exposed HBEC, a model of the earliest alterations in the progression of lung malignancy, Fzd9 expression changes correlate with the 1 week smoke mouse model where increased prostacyclin in whole lung tissue led to elevated Fzd9 expression.

In an effort to identify a target of iloprost that might be useful as an efficacy biomarker and also provide a mechanism for changes in Fzd9 expression, we investigated miRNA changes after exposure to prostacyclin. miR-31 increased with exposure to cigarette smoke and decreased with lung specific prostacyclin overexpression or iloprost treatment. Fzd9 expression was inversely related to miR-31 expression *in vitro* and *in vivo* and decreased with transfection of a miR-31 mimic regardless of CSC or iloprost exposure. Previous work has shown that miR-31 expression is associated with lung cancer progression in humans and with KRAS tumors in mice^23^,^24^,^25^,^26^. It is also involved in regulation of lung cancer stem-like cells^27^. miR-31 was proposed as a biomarker for drug activity in a murine lung cancer chemoprevention model using vinyl carbamate and indole-3-carbinol^28^. Evidence for the technical feasibility of miR-31 marker detection in surrogate biospecimens was presented using digital PCR to analyze expression in sputum^29^. miR-31 may be useful as a biomarker for monitoring iloprost activity in surrogate specimens as an alternative to biopsy by bronchoscopy during chemoprevention treatment.

miR-31 is not strongly predicted to directly target Fzd9 based on analysis by several programs, however, as target prediction can be unreliable, future studies will include Fzd9 3′UTR reporter analysis to experimentally test the interaction^30^,^31^,^32^. Regulation of Fzd9 expression is poorly understood, but if we find that miR-31 does not directly regulate Fzd9, known targets of miR-31 could provide direction for future studies of Fzd9 regulation. miR-31 has many predicted targets, including hub genes PIK3CA, JUN, MAPK1, MAPK3, CCND1, FOS, MDM2, KRAS, EGFR, PTK2, and VEGFA^33^. In the area of WNT signaling, miR-31 stimulates canonical Wnt/β-catenin by depleting repressors of the pathway and increases expression of non-canonical Wnt5a, which induces EMT^22^. Further studies are needed to determine whether other WNT pathway components, such as Dkk or SFRP1, are targeted by miR-31 or involved in iloprost stimulated Fzd9 signaling.

We demonstrated that exposure to cigarette smoke represses Fzd9 expression and since Fzd9 is required for iloprost activation of PPARγ, this could explain the lack of response in current smokers in the oral iloprost clinical chemoprevention trial. If Fz9d expression increases with smoking cessation, this could explain the range of response seen in former smokers in the trial, which may be dependent on quit time. Our *in vitro* data supports the testing of Fzd9 as a biomarker of response in tissues from the iloprost chemoprevention trial and potentially using Fzd9 as an eligibility criterion for future trials. If we can establish Fzd9 as a predictive marker of response to iloprost, future clinical trials will have better targeted subject pools, which will decrease cost, decrease sample size, and prevent unnecessary treatments. Increased expression of Fzd9 with prostacyclin suggests that Fzd9 could also be a marker for activity of iloprost. In combination with surrogate samples analysis, such as sputum, this could greatly alleviate the time, cost, and use of an invasive procedure (bronchoscopy) for endpoint analysis in patients. Data from this study has provided valuable preclinical data supporting clinical implementation of lung cancer chemoprevention. Our group recently began a Phase I trial using inhaled iloprost. The oral iloprost trial has and this trial will have specimens available for validation of Fzd9 as a prediction and response marker. This work demonstrates the value of “bedside to bench” translational research, where clinical data motivates biomarker and mechanistic studies that have immediate clinical relevance.

## Methods

### Cell Culture

Human bronchial epithelial cells (HBEC) (A gift from Dr. John Minna) were cultured in Keratinocyte Serum Free Medium (GIBCO) at 37 °C in a humidified 5% CO_2_ incubator and passaged twice per week. Primary dysplastic lung cell cultures isolated from biopsies at the University of Colorado Cancer Center were grown in Bronchial Epithelial Basal Media (Lonza) at 37 °C in a humidified 5% CO_2_ incubator. Morphology of cells lines was verified twice weekly. Cigarette smoke condensate (CSC) (Murty Pharmaceuticals) was applied at 5 ug/mL concentration and Iloprost (Cayman Chemicals) at 10 uM in cell growth media. Cells were allowed 24 hours for recovery after plating before drug or carcinogen application. HBEC with CSC and iloprost exposure were carried in triplicate as individual cell lines with twice a week passaging for 1–24 weeks. DMSO was used for a vehicle control. All long-term HBEC cultures were grown and handles in a dedicated incubator and biosafety cabinet.

### Human Tissues

The University of Colorado Cancer Center SPORE in Lung Cancer Tissue Bank and Biomarkers Core (Tissue Bank) provides sets of publicly available, deidentified samples for small studies. The Colorado Multiple Institutional Review Board approves all the human experimental protocols that generate specimens for the Tissue Bank and collects written, informed consent from all subjects. RNA from 17 pairs of deidentified matched human normal lung and NSCLC tumor tissues were obtained from the Tissue Bank after application to the Tissue Use Committee.

### Mouse Tissues

All procedures were approved by the Denver Veterans Administration Medical Center institutional animal care and use committee. Mouse tissues used in this study were previously generated and frozen in RNALater (Qiagen). For urethane studies, wild type AJ or wildtype and prostacyclin synthase transgenic (PGIStg) FVB mice, one 1 mg/g urethane i.p. injection was given and mice were sacrificed 20 weeks later^11^. Tumors and uninvolved tissue were dissected for RNA extraction. For the one-week smoke exposure study, wild type and PGIStg FVB mice were exposed to whole body cigarette smoke in a TE-10 smoking machine (Teague Enterprises) at 80 mg/m^3 ^3. Mice were sacrificed after one week and whole lung tissue was removed for RNA extraction. All experiments were conducted in accordance with AAALAC International guidelines and the United State Animal Welfare Act.

### Quantitative PCR

RNA was extracted from HBEC and dysplastic cells using the AllPrep Universal kit (Qiagen) and from mouse tissue using the AllPrep DNA/RNA kit (Qiagen) on the Qiacube automated processor (Qiagen). mRNA was reverse transcribed using the ABI HC cDNA kit (Thermo Fisher Scientific) and miRNA using the miScript II RT Kit (Qiagen). qPCR primers included: hu and ms Fzd9, hu and ms GAPDH, ms 18sRNA, hu Snail, hu Ecadherin, hu Vimentin, hu Crumbs3, and hu Zona Occludens-1 mRNA (Bio-Rad) and for hsa-miR-31 and RNU6 miRNA (Qiagen). qPCR was conducted using standard protocols for Sso Advanced SYBR Green Master Mix (Bio-Rad) or the miScript SYBR Green PCR Kit (Qiagen) on a CFX96 Touch (Bio-Rad). Genes for normalization of mouse model PCR were determined by screening 8 genes with the Mouse Housekeeping Genes PCR Array (Qiagen, PAMM-000Z). All PCR reactions were conducted in triplicate.

### miRNA PCR Array

For initial miRNA screening, HBEC cells were treated with 24 weeks CSC followed by 4 weeks with DMSO or iloprost. Total RNA was extracted and reverse transcribed with a miScript II RT kit (Qiagen). miRNA expression was screened using a miScript miRNA PCR Array focused on cancer stem cells (Qiagen, MIHS-118Z). The array profiled expression of 84 miRNAs and included a set of six normalization controls as well as controls to assess RNA recovery, reverse transcription performance, and PCR performance. Results were analyzed through the SABiosciences data analysis portal.

### Transfections

HBEC cells were cultured with iloprost for 6 weeks. Cells were then transfected with hsa-miR-31 mimic (2.5 nM) (Qiagen) or a negative control (2.5 nM) and 1.1 ul TransIT-X2 (Mirus Bio) per well in 24-well plates. After 48 hours, total RNA was extracted for qPCR analysis. Transfections were done in duplicate.

### Statistical Analysis

For single comparisons, a one-sided t-test was used to calculate p-values. For multiple comparisons, a planned comparison approach was used^34^. Multiple comparisons to a single control employed Dunnett’s multiple comparison test. Comparisons between multiple means employed Fishers Least Significant Difference test. As a result, p-values are not corrected for multiple comparisons. p-values greater than 0.05 but less than 0.1 were not considered significant but are listed in the figure legends. All statistical analysis was done using GraphPad Prism software.

## Additional Information

**How to cite this article**: Tennis, M. A. *et al*. Prostacyclin reverses the cigarette smoke-induced decrease in pulmonary Frizzled 9 expression through miR-31. *Sci. Rep*. **6**, 28519; doi: 10.1038/srep28519 (2016).
